# Naphthalene Monoimides with *Peri*-Annulated Disulfide Bridge—Synthesis and Electrochemical Redox Activity

**DOI:** 10.3390/ma16237471

**Published:** 2023-12-01

**Authors:** Monika Mutovska, Natali Simeonova, Stanimir Stoyanov, Yulian Zagranyarski, Silva Stanchovska, Delyana Marinova

**Affiliations:** 1 Faculty of Chemistry and Pharmacy, Sofia University “St. Kliment Ohridski”, 1164 Sofia, Bulgaria; ohmgm@chem.uni-sofia.bg (M.M.); natali.n.simeonova@gmail.com (N.S.); sstoyanov@chem.uni-sofia.bg (S.S.); 2 Institute of General and Inorganic Chemistry, Bulgarian Academy of Sciences, 1113 Sofia, Bulgaria; stanchovska@svr.igic.bas.bg

**Keywords:** organosulfur electrode materials, *n*- and *p*-type redox reactions, dithiole heterocycle annulation, 1,8-naphthalimide derivatives

## Abstract

Nowadays, organosulfur compounds provide new options in the development of full organic ion batteries. However, many drawbacks (such as kinetics limitations during the reversible oxidation of disulfides with cleavage of S–S bond, as well as solubility in non-aqueous electrolytes) make their commercialization difficult. Herein, a new concept for the design of organosulfur compounds with regulated redox properties and limited solubility is proposed. As a proof-of-concept, we designed *peri*-disulfo-substituted 1,8-naphthalimide derivatives, in which the alkyl chain length and halogen substituents (Cl or Br) at positions 3 and 6 are varied. The compounds were synthesized by an originally developed procedure starting from tetrahalonaphthalic anhydride via nucleophilic substitution at both *peri*-positions in the respective imide. Using ionic liquid electrolyte, it was found that the new *peri*-dithiolo-1,8-naphthalimides can participate in *n*- and *p*-type redox reactions at about 2.0 V and above 4.0 V vs. Li/Li^+^, respectively. The redox potentials are sensitive mainly to whether Cl or Br substituents are available in the molecule architecture, while the alkyl chain length determines the kinetics of the redox reactions. Among all compounds, the chloro-substituted compound with the shorter alkyl chain displays the best kinetics for both low- and high-voltage redox reactions.

## 1. Introduction

Lithium-ion batteries (LiIBs) are nowadays indispensable devices for mobile energy storage [1,2]. They function thanks to the reversible redox reactions of Li^+^ ions with the two electrodes [1,2,3]. Although LiIBs offer the highest energy density, they are in disconformity with strong environmental requirements since the key ingredient of electrode materials is the toxic and expensive Co element [4,5]. The cobalt content in widely used batteries (i.e., those worked with layered LiCoO_2_ and LiCo_x_Ni_y_MnzO_2_ electrodes) varies in the range of 0.05–0.37 kg Co per kW h [6]. Depending on how the spent batteries are disposed, Co can leach out from batteries as ions, which are easily accumulated in natural waters, lands, plants and crops, thus provoking problems for human health and all living organisms [7]. The toxicity of Co also encompasses the release of oxide nanomaterials (such as layered LiCoO_2_ and LiCo_x_Ni_y_Mn_z_O_2_ electrodes), which become dangerous for humans and wildlife if they are breathed [7].

In searching for greener electrodes without compromising their energy density, redox organic materials emerge as an alternative to conventional inorganic materials due to their greater variety of compositions and redox reaction mechanisms, a finer structure design and easier accommodation of structural stresses occurring during the redox reactions, a weaker dependence of the redox properties from the ionic sizes, and charges of the metal ions participating as oxidizers or reducers [8,9].

Among organic materials, organopolysulfide compounds can be singled out because of their unique redox properties [10,11,12]. The reversible redox reaction of Li^+^ with organopolysulfide compounds has been shown to proceed with cleavage and subsequent formation of the sulfur–sulfur (S–S) bond: RS–SR + 2Li^+^ +2e^−^↔2RSLi [13]. Several types of organic groups bonded to sulfur bridge have been examined, starting from aromatic molecules reaching to polymers [14,15]. Irrespective of the variety of organosulfur compositions (i.e., in terms of the linkage of the sulfur with organic groups and the number of sulfur chain [10]), the two-electron redox reaction of Li^+^ with the (S–S) bond enables one to reach a high specific capacity, but at the expense of the slow kinetics. Furthermore, the organic electrode materials suffer from their relatively high dissolution in the electrolyte, as well as their instability during electrochemical reaction [13]. There are two approaches to overcoming these drawbacks: (i) functionalization or doping of organosulfur compounds; and (ii) finding a suitable electrolyte. Through the functionalization of phenyl disulfide on CNT, both kinetics and reversibility of the redox reaction are significantly enhanced [16]. The addition of *N*-heterocycles into organosulfur compounds enables a better control on the redox properties; for example, after the substitution of two carbons with N atoms in dipyridyl disulfide, the discharge voltage increases from 2.2 to 2.45 V together with the improvement of the cycling stability [17]. The most utilized electrolytes encompass liquids ones, such as solutions of the given lithium salt (predominately LiClO_4_ or LiTFSI) into carbonate-based solvent [13].

Although the redox reaction of the polysulfide moieties proceeds with the participation of Li^+^ ions (i.e., *n*-type redox reaction), the organosulfur compounds display a *p*-type redox reactivity, according to which the organic fragment is first oxidized and is then bound to electrolyte anion. The main representatives of the *p*-type compounds are the thioethers [13]. In general, the *p*-type redox reaction is usually accomplished at higher redox potentials than that of the *n*-type. Because of the different mechanisms, both *n*- and *p*-type reactions are characterized with different kinetics and capacity. However, the main drawback concerning the solubility of organosulfur compounds in electrolytes remains for *p*- and *n*-type compounds. Therefore, the state-of-the-art studies have mainly focused on finding organosulfur compounds with improved properties.

Among the classes of functional organic compounds, the 1,8-naphthalen monoimides (NMIs) and diimides (NDIs) have attracted substantial scientific interest due to their excellent chemo- and photostability, accessibility, and relatively easy derivatization capabilities. Core-substituted NDIs have found numerous applications in organic electronics and electrochemistry, for example, as field effect transistors [18] and new cathode materials for LiIBs [19]. Typically, they undergo reversible redox reactions at E_1/2_ = −1.10 V and E_1/2_ = −1.51 V vs. Fc/Fc+ (or E_1/2_ = 2.32 V and E_1/2_ = 1.91 V expressing in Li/Li^+^ scale) [20]. The presence of electron-withdrawing imide fragments in the NMI structure allows for straightforward substitution at the *peri*-positions (4 and 5) with various nucleophiles, thus forming donor–π–acceptor type systems with interesting electronic properties due to the intramolecular charge transfer (ICT) in their molecules. The push–pull effect leads to a drastic decrease in the HOMO-LUMO transition energy, manifested in a strong bathochromic shift of the absorption and fluorescence bands with respect to the unsubstituted NMIs. The desired photophysical properties allowed for their successful application as OLEDs [21], optical sensors [22], fluorescent cellular imaging agents, and DNA targeting binders [23], etc. Concerning electrochemical energy storage, the organic charge transfer complexes have been proposed as a new approach for improving the electrical conductivity and dissolution of redox-active organic compounds [24].

**Figure 1:**
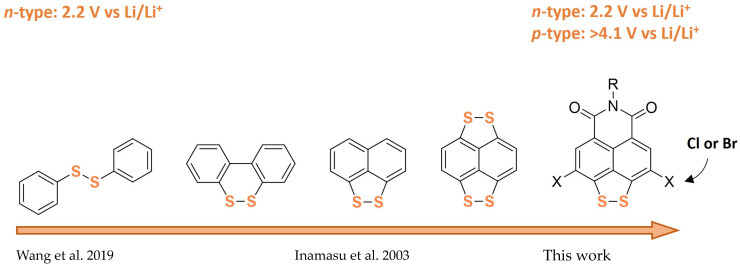
Evolution of disulfide cathode materials [17,26].

This study aims to propose a new concept for design of organosulfur compounds with regulated redox properties and limited solubility. As a proof-of-concept, we designed *peri*-disulfo-substituted 1,8-naphthalimide derivatives, in which the alkyl chain length and halogen substituents (Cl or Br) at positions 3 and 6 are varied. Thus, designed *peri*-dithiolo annulated 1,8-naphthalimides are compared with previously reported organosulfur compounds (Figure 1). It is worth mentioning that the redox properties of complexes of iron with naphthalene monoimide derivatives of *peri*-substituted dichalcogenides have been already examined in aqueous solutions [25]. As far as we know, there are no data on the redox properties of *peri*-dithiolo-1,8-naphthalimide compounds in non-aqueous lithium electrolytes. Therefore, the first part of the study is focused on the synthesis of these compounds. The second part of the study covers their redox properties, which are examined in model Li-ion cells using ionic liquid as an electrolyte. The used methodology allows us to monitor the effects of the halogen substituents and alkyl chain length on the redox properties of the new *peri*-dithiolo-1,8-naphthalimides.

## 2. Materials and Methods

All starting materials (excepting the synthesized by us) and solvents were commercially available and used without additional purification (Merck (Darmstadt, Germany), Fisher Scientific (Hampton, NH, USA), Fluorochem (Glossop, UK), Sigma-Aldrich (St. Louis, MO, USA)). Thin-layer chromatography was used to monitor the progress of all reactions (Macherey-Nagel F 254 silica gel sheet, Macherey-Nagel, Duren, Germany) using appropriate mixture of solvents as eluent (described for each compound in the synthetic procedures). Column chromatography on silica gel (Macherey Nagel, 0.063 mm–0.200 mm) was used for purification. NMR spectra were recorded on a Bruker Avance 500 MHz instrument (Bruker, Karlsruhe, Germany). Spectra (^1^H, ^13^C{1H}) were referenced to appropriate residual solvent signals (CDCl_3_, C_2_D_2_Cl_4_). Elemental analyses were carried out on a Leco CHNS-932 (Leco Europe, Geleen, The Netherlands).

### 2.1. Synthesis

#### 2.1.1. Synthesis of (*N*-octyl)-3,4,5,6-tetrahalo-1,8-naphthalimides **3** and **4**

**General procedure:** The corresponding 1,8-naphthalic anhydride **1** or **2** (50.0 mmol) was suspended in a mixture of 150 mL NMP and 150 mL acetic acid. After *n*-octylamine (1.5 eq, 75.0 mmol, 9.70 g) was added, the resulting mixture was stirred for 1 h at 110 °C then cooled down slowly to room temperature and afterwards poured into ice. The formed precipitate was filtered, washed thoroughly with water, and dried. The crude product was purified by column chromatography on silica (hexane/dichloromethane as eluent) to afford the target compounds as a slightly yellowish solid.

##### Synthesis of (*N*-octyl)-3,4,5,6-tetrachloro-1,8-naphthalimide **3**

Yield after column chromatography 20.80 g (93%).

^1^H-NMR (δ (ppm), CDCl_3_): 0.87 (t, 3H, CH_3_, *J* = 7.0 Hz); 1.23–1.42 (m, 10H, CH_2_); 1.69 (p, 2H, CH_2_, *J* = 7.5 Hz); 4.11–4.14 (m, 2H); 8.65 (s, 2H).

^13^C-NMR (δ (ppm), CDCl_3_): 14.23; 22.77; 27.16; 28.04; 29.31; 29.31; 29.40; 31.39; 41.15; 122.43; 127.75; 128.96; 132.94; 135.37; 137.14; 161.96.

Anal. calcd. C_20_H_19_Cl_4_NO_2_: C, 53.72; H, 4.28; N, 3.13; Found: C, 53.55; H, 4.01; N, 3.27.

##### Synthesis of (*N*-octyl)-3,4,5,6-tetrabromo-1,8-naphthalimide **4**

Yield after column chromatography 28.12 g (90%).

^1^H-NMR (δ (ppm), CDCl_3_): 0.87 (t, 3H, CH_3_, *J* = 6.9 Hz); 1.27–1.41 (m, 10H, CH_2_); 1.69 (p, 2H, CH_2_, *J* = 7.5 Hz); 4.10–4.13 (m, 2H); 8.78 (s, 2H).

^13^C-NMR (δ (ppm), CDCl_3_): 14.23; 22.77; 27.15; 28.04; 29.31; 29.40; 31.39; 41.12; 122.91; 127.87; 129.59; 131.38; 132.73; 135.78; 162.03.

Anal. calcd. C_20_H_19_Br_4_NO_2_: C, 38.44; H, 3.06; N, 2.24; Found: C, 38.31; H, 2.99; N, 1.95.

#### 2.1.2. Synthesis of (*N*-butyl)-3,4,5,6-tetrahalo-1,8-naphthalimides **5** and **6**

**General procedure:** The corresponding 1,8-naphthalic anhydride **1** or **2** (50.0 mmol) was suspended in a mixture of 200 mL NMP and 200 mL acetic acid. After *n*-butylamine (1.5 eq, 75.0 mmol, 5.49 g) was added the resulting mixture was stirred for 2 h at 110 °C then cooled down slowly to room temperature and afterwards poured into ice. The formed precipitate was filtered, washed thoroughly with water, and dried. The crude product was purified by column chromatography on silica (hexane/dichloromethane as eluent) to afford the target compounds as pale gray solid.

##### Synthesis of (*N*-butyl)-3,4,5,6-tetrachloro-1,8-naphthalimide **5**

Yield after column chromatography 17.79 g (91%).

^1^H-NMR (δ (ppm), CDCl_3_): 0.97 (t, 3H, CH_3_, *J* = 7.4 Hz); 1.42 (h, 2H, CH_2_, *J* = 7.4 Hz); 1.69 (p, 2H, CH_2_, *J* = 7.6 Hz); 4.12–4.15 (m, 2H); 8.65 (s, 2H).

^13^C-NMR (δ (ppm), CDCl_3_): 13.91; 20.41; 30.10; 40.89; 122.42; 127.74; 128.95; 132.94; 135.38; 137.14; 161.97.

Anal. calcd. C_16_H_11_Cl_4_NO_2_: C, 49.14; H, 2.84; N, 3.58; Found: C, 48.88; H, 2.61; N, 3.29.

##### Synthesis of (*N*-butyl)-3,4,5,6-tetrabromo-1,8-naphthalimide **6**

Yield after column chromatography 25.03 g (88%).

^1^H-NMR (δ (ppm), CDCl_3_): 0.97 (t, 3H, CH_3_, *J* = 7.3 Hz); 1.42 (h, 2H, CH_2_, *J* = 7.4 Hz); 1.68 (p, 2H, CH_2_, *J* = 7.6 Hz); 4.12–4.15 (m, 2H); 8.79 (s, 2H).

^13^C-NMR (δ (ppm), CDCl_3_): 13.91; 20.41; 30.12; 40.87; 122.91; 127.88; 129.61; 131.38; 132.75; 135.79; 162.06.

Anal. calcd. C_16_H_11_Br_4_NO_2_: C, 33.78; H, 1.95; N, 2.46; Found: C, 33.98; H, 1.63; N, 2.45.

#### 2.1.3. Synthesis of 6-Alkyl-3,9-dihalo-5*H*-[1,2]dithiolo[3′,4′,5′:4,5]naphtho[1,8-cd]pyridine-5,7(6*H*)-diones **7**–**10**

**General procedure:** A mixture of the corresponding 3,4,5,6-tetrahalo-1,8-naphthalimide (10.0 mmol) and sulfur (2 eq, 40.0 mmol, 1.28 g) in 50 mL NMP was stirred for 4–5 h at 175 °C. The mixture was slowly cooled down to room temperature and poured into ice. The formed precipitate was filtered, washed thoroughly with water, and dried. The crude product was purified via column chromatography on silica (hexane/dichloromethane as eluent) or recrystallized from dioxane to afford the target compounds as a yellow solid.

##### Synthesis of 3,9-Dichloro-6-octyl-5*H*-[1,2]dithiolo[3′,4′,5′:4,5]naphtho[1,8-cd]-pyridine-5,7(6*H*)-dione **SCl8**

Yield after column chromatography 4.10 g (93%).

^1^H-NMR (δ (ppm), CDCl_3_): 0.87 (t, 3H, CH_3_, *J* = 6.8 Hz); 1.27–1.42 (m, 2H, CH_2_); 1.68 (p, 2H, CH_2_, *J* = 7.5 Hz); 4.11–4.14 (m, 2H, CH_2_); 8.32 (s, 2H).

^13^C-NMR (δ (ppm), CDCl_3_): 14.24; 22.78; 27.25; 28.13; 29.36; 29.47; 31.95; 40.97; 119.67; 123.28; 126.47; 131.98; 134.59; 150.34; 162.12.

Anal. calcd. C_20_H_19_Cl_2_NO_2_S_2_: C, 54.55; H, 4.35; N, 3.18; Found: C, 54.27; H, 4.18; N, 3.35.

##### Synthesis of 3,9-Dibromo-6-octyl-5*H*-[1,2]dithiolo[3′,4′,5′:4,5]naphtho[1,8-cd]-pyridine-5,7(6*H*)-dione **SBr8**

Yield after column chromatography 4.76 g (90%).

^1^H-NMR (δ (ppm), CDCl_3_): 0.87 (t, 3H, CH_3_, *J* = 6.8 Hz); 1.23–1.42 (m, 2H, CH_2_); 1.68 (p, 2H, CH_2_, *J* = 7.5 Hz); 4.11–4.14 (m, 2H, CH_2_); 8.43 (s, 2H).

^13^C-NMR (δ (ppm), CDCl_3_): 14.24; 22.79; 27.25; 28.13; 29.36; 29.47; 31.96; 40.96; 110.55; 119.45; 127.12; 133.39; 134.50; 153.49; 161.98.

Anal. calcd. C_20_H_19_Br_2_NO_2_S_2_: C, 45.38; H, 3.62; N, 2.65; Found: C, 45.14; H, 3.88; N, 2.89.

##### Synthesis of 6-Butyl-3,9-dichloro-5*H*-[1,2]dithiolo[3′,4′,5′:4,5]naphtho[1,8-cd]-pyridine-5,7(6*H*)-dione **SCl4**

Yield after column chromatography 3.65 g (95%).

^1^H-NMR (δ (ppm), CDCl_3_): 0.98 (t, 3H, CH_3_, *J* = 7.3 Hz); 1.42 (h, 2H, CH_2_, *J* = 7.3 Hz); 1.67 (p, 2H, CH_2_, *J* = 7.7 Hz); 4.13 (t, 2H, *J* = 7.5 Hz); 8.33 (s, 2H).

^13^C-NMR (δ (ppm), CDCl_3_): 13.85; 20.26; 29.90; 40.46; 119.20; 122.96; 126.21; 131.74; 134.38; 150.34; 161.89.

Anal. calcd. C_16_H_11_Cl_2_NO_2_S_2_: C, 50.01; H, 2.89; N, 3.64; Found: C, 49.78; H, 2.99; N, 3.85.

##### Synthesis of 3,9-Dibromo-6-butyl-5*H*-[1,2]dithiolo[3′,4′,5′:4,5]naphtho[1,8-cd]-pyridine-5,7(6*H*)-dione **SBr4**

Yield after recrystallization 4.45 g (94%).

^1^H-NMR (δ (ppm), CDCl_3_): 0.97 (t, 3H, CH_3_, *J* = 7.3 Hz); 1.42 (h, 2H, CH_2_, *J* = 7.4 Hz); 1.67 (p, 2H, CH_2_, *J* = 7.9 Hz); 4.13 (t, 2H, *J* = 7.6 Hz); 8.48 (s, 2H).

^13^C-NMR (δ (ppm), CDCl_3_): 14.85; 21.26; 30.91; 41.45; 111.29; 120.01; 127.93; 134.26; 135.31; 154.55; 162.80.

Anal. calcd. C_16_H_11_Br_2_NO_2_S_2_: C, 40.61; H, 2.34; N, 2.96; Found: C, 40.38; H, 2.11; N, 3.17.

### 2.2. Electrochemical Characterization

The cycling voltammetry experiments were carried out using Swagelok-type three-electrode cells. The positive electrode consists of a mixture between *peri*-substituted disulfides (**SCl4**, **SCl8**, **SBr4**, or **SBr8**), 10% Super C65 (TIMCAL), and 10% sodium carboxymethyl cellulose (CMC) in a ratio 80-10-10 wt%. The slurry was spread on an aluminum foil, double side carbon-coated, and dried overnight at 80 °C. Disks with a diameter of 10 mm were cut and additionally dried under vacuum. The active mass loaded on Al collectors was about 2–3 mg. The electrolyte for the electrochemical tests was a 1 M LiTFSI-Pyr_1,3_FSI (Lithium bis (trifluoromethanesulfonyl) imide in *N*-methyl, propyl pyrrolidinium bis (fluorosulfonyl) imide) 1:9 by ratio). This electrolyte is chosen over the conventional carbonate-based electrolyte (i.e., 1 M LiPF_6_ in EC/DMC) due to the insolubility of the organosulfur compounds.

The assembly of the cells was carried out in an MB-Unilab glovebox model Pro SP (1500/780), with a low content of moisture and oxygen (under 0.1 ppm). The electrochemical reactions were performed in potentiostatic mode on a multi-channel potentiostat/galvanostat Biologic VMP-3e including impedance meter. The voltage window for cycling of model lithium half-cells was 1.5–5.0 V and scan rates were between 100 mV/s and 1 mV/s.

## 3. Results and Discussion

### 3.1. Design Concept

Despite being one of the most promising electrode materials and the subject of enormous scientific interest, disulfide compounds suffer from several unsolved problems: (i) Poor recombination (oxidation) of dithiolate ions back to disulfide due to particle diffusion or rotation in the cyclic compounds; (ii) the LUMO levels are usually too high, and the molecule does not undergo cleavage of the two-electron sulfide bond but rather a reversible one-electron reduction [27]; (iii) problems with the solubility of the electrode material [28,29].

**Figure 2:**
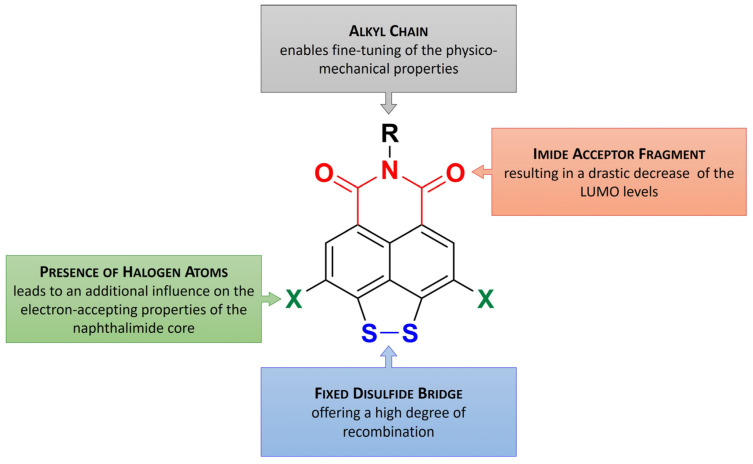
Design of target compounds.

To tackle these limitations, we designed the bridged *peri*-disilfo-substituted 1,8-naphthalimide core structure shown on Figure 2. The main advantage of this architecture is the fixed dichalcogenide bridge, offering a high degree of recombination and hence good electrochemical reversibility. Furthermore, the dithiole cycle is aromatic, which makes its reconstruction even more favorable. Another benefit is the presence of an imide acceptor fragment, perfectly positioned *peri* to the disulfide bridge, which should lead to a drastic decrease in the LUMO levels and hence a significantly easier progress of the reduction process. Additionally, it is possible to fine-tune the physical and mechanical properties (solubility, crystallization ability, thermo- and chemostability, etc.) by varying the type and length of the alkyl chain at one hand, as well as the electron-accepting properties of the naphthalimide core due to the presence of different halogen atoms in positions 3 and 6 on the other.

**Figure 3:**
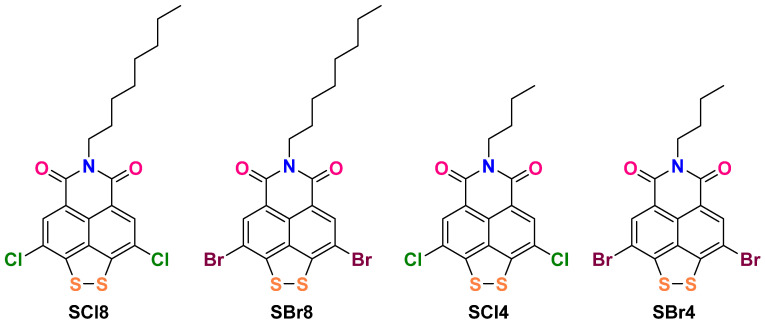
Structures of target compounds.

Based on the design concept, the following target compounds were planned for synthesis (Figure 3).

### 3.2. Synthesis

**Scheme 1:**
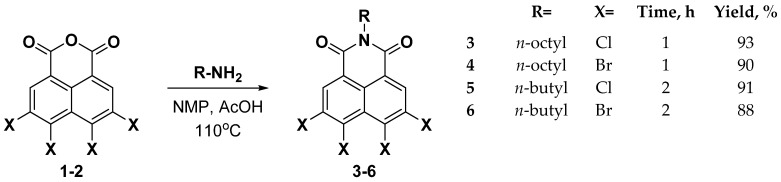
Synthesis of imides **3**–**6**.

Tetrahaloanhydrides **1** and **2** were identified as suitable starting compounds and were prepared according to a previously published procedure [25]. The first step was the synthesis of the corresponding naphthalimides **3**–**6** (Scheme 1). The higher nucleophilicity of the aliphatic amines and the good solubility of the tetrahaloanhydrides allowed the imidization to be carried out at relatively low temperatures compared to those previously described [25,30]. The imidization of **1** and **2** with *n*-octylamine was carried out in a mixture of *N*-methylpyrrolidone (NMP) and acetic acid at 110 °C for 1 h. After workup and purification via column chromatography, we isolated 3,4,5,6-tetrachloro-1,8-naphthalimide **3** and 3,4,5,6-tetrabromo-1,8-naphthalimide **4** with excellent yields (93% and 90%, respectively). Both *N*-(*n*-octyl) substituted imides are very soluble in organic solvents, making them suitable as starting compounds for the formation of a dithiole ring with the participation of the activated halogen atoms at positions 4 and 5.

It is known that the chain length and branching are the two most important properties of the *N*-alkyl substituents in the naphthalimides, and they have a huge impact on their solubility. In general, they decrease dramatically when short and straight alkyl chains are used [31,32]. To test the solubility limits of the target disulfide compounds, we aimed to synthesize analogues of the *n*-octyl derivatives with shorter alkyl chain. For this purpose, we also reacted **1** and **2** with *n*-butylamine under similar conditions (Scheme 1) and successfully isolated the imides **5** and **6** in very high yields (91% and 88%, respectively), although in twice as long reaction time. After purification by column chromatography, imides **3**–**6** were characterized via ^1^H and ^13^C-NMR and elemental analysis. It is important to note that the crude imides **3**–**6** can also be easily purified by a simple reprecipitation procedure. The crude substances are dissolved in a minimal amount of dichloromethane, filtered, and reprecipitated with a large excess of methanol. Thus, they can be obtained in very high purity and used in the next step directly. Yields are reduced by only 3–5%, which makes this method very convenient.

**Scheme 2:**
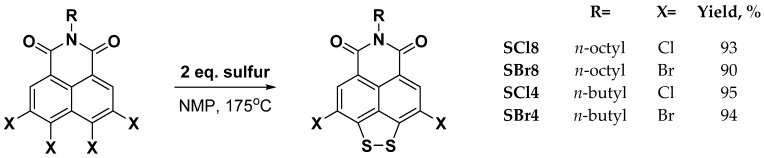
Synthesis of dithiolo-annulated naphthalimides.

The next step in our synthetic strategy was the formation of the new five-membered ring with two S-atoms bonded at positions 4 and 5 of the naphthalimides (Scheme 2). Based on our previous studies [25], we first attempted optimization of the synthetic procedure for this key step, both in terms of reaction conditions and scale. As a model reaction for dithiole heterocycle formation, we used imide **3**. The reaction yields were improved by slightly raising the temperature up to 175 °C and reducing the amount of sulfur to 2.5-fold compared to our previously reported similar reaction. This also resulted in significantly shorter reaction times, which were decreased by 40 to 50%. The reaction was further tested with respect to the use of an inert atmosphere, and the yield was found to practically not change when carried out under air or argon. To test the scale-up potential, after the optimization the reaction was carried out in 10, 20, and 40 mmol scales, yields of 93, 92, and 87% were achieved, respectively, which suggests promising scalability, with just a slight decrease of 1 to 6%, when increasing the scale by a factor of two and four, respectively.

The optimized conditions were then applied to the rest of the tetrahalogen-substituted imides also. The reaction between **4** and sulfur afforded the final product, **SBr8**, in high yield (90%) on a gram scale. The *N*-(*n*-butyl)-substituted imides **5** and **6** also reacted with sulfur under the same conditions as their *n*-octyl analogues with even higher yields (Scheme 2). In this case, only imide **SCl4** had sufficient solubility to allow for purification via column chromatography. The other target product, **SBr4**, was purified via recrystallization from dioxane. All final products were obtained in very high yields and purity and have been characterized by means of ^1^H, ^13^C{1H}-NMR spectroscopic techniques, and elemental analysis (see Section 2 and Supplementary Materials).

### 3.3. Redox Properties of Peri-Substituted Dichalchogenides

**Figure 4:**
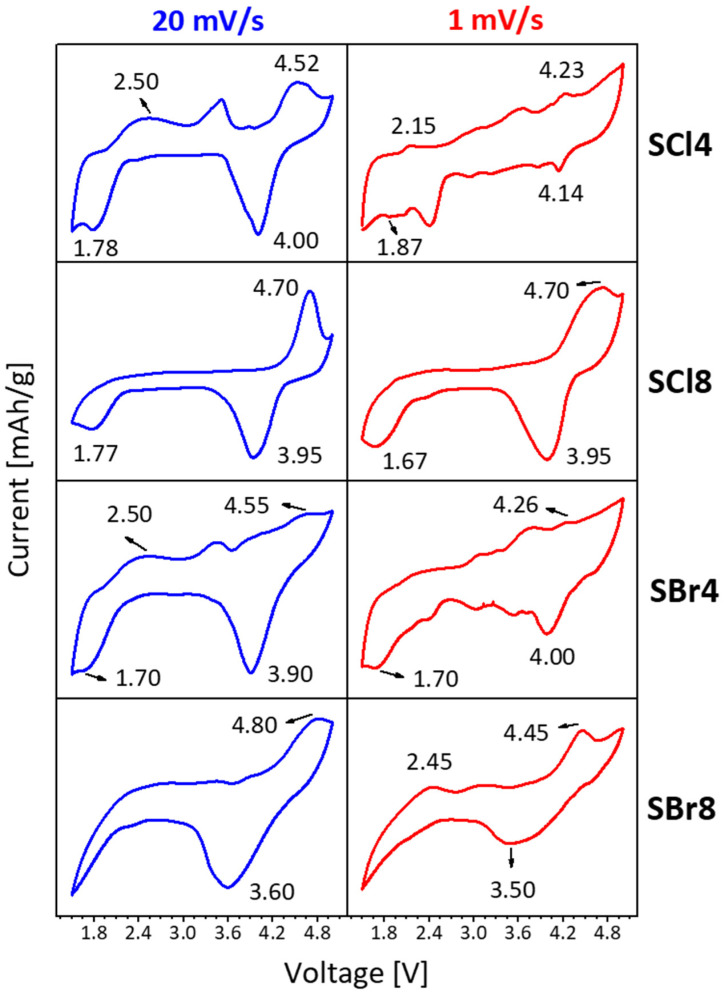
CV curves in a broad potential range (1.5–5.0 V) at a scanning rate of 20 mV/s (**left**) and 1 mV/s (**right**) for **SCl4**, **SCl8**, **SBr4**, and **SBr8**. As an electrolyte, ionic liquid comprising 1M LiTFSI-Pyr_1,3_FSI solution is used.

Figure 4 compares the CV curves of *peri*-dithiolo-naphthalimides. The CV curves are recorded in a broad potential range (i.e., between 1.5 and 5.0 V) due to the stability of the electrolyte based on ionic liquids [33]. For all compositions, two redox bands can be distinguished in low- (i.e., below 2.5) and high-voltage (i.e., above 4.0 V) regions of the CV curves, respectively. The comparison of the CV curve profiles demonstrates that the low-voltage redox reaction proceeds more easily for compounds with shorter alkyl chain (**SCl4** and **SBr4**), while the longer alkyl chain gives rise to the high-voltage redox reaction. In comparison with the alkyl chain length, the presence of Cl and Br atoms at positions 3 and 6 predominantly affects the magnitude of the redox potential: going from Cl to Br substituents, the redox potential (expressed by E_1/2_ measured at a scan rate of 20 mV/s) decreases from 4.26 V to 4.22 V and from 2.14 V to 2.10 V for **SCl4** and **SBr4**, while for **SCl8** and **SBr8**, the decrease is from 4.33 V to 4.20 V, respectively (the low-voltage band is not well resolved for these compounds).

Taking into account the previous data on the redox properties of organosulfur compounds [34], the low-voltage band can be associated with a redox reaction occurring between Li^+^ ions and the disulfide moiety of the organic compounds. It is worth mentioning that for a simple organosulfur compound such as diphenyl disulfide, the potential of Li+ interaction is of 2.20 V [17], which approaches the experimentally determined one for **SCl4**. However, the possible participation of the attached –C=O groups in redox reaction with Li^+^ cannot be excluded [35]: for example, the unsubstituted naphthalene diimides interact with 2 Li^+^ at a potential of 2.55 V, which is much higher than that determined for **SCl4** (2.14 V). This signifies that the low potential reaction of **SCl4** with Li^+^ takes place predominantly with the participation of disulfide bridge. According to the well-accepted classification of the organic redox compounds [36], the low-voltage reaction of *peri*-substituted compounds can be categorized as *n*-type ones.

Contrary to the low-voltage band, the high-voltage band could be attributed to the oxidation of the *peri*-substituted disulfides followed by bonding with anions from the electrolyte (TFSI^−^). This reaction can be classified as a *p*-type. It is noticeable that for the *p*-type organic compounds, the highest potentials have so far been reported for phenazine-derived compounds, in which *N*-atoms are replaced by S and O: for example, 4.1 V for thianthrene and 4.2 V for dibenzodioxin, respectively [24,37]. The comparison shows that **SCl8** prepared by us displays even higher potential (i.e., E_1/2_ = 4.3 V at a scan rate of 1 mV/s, Figure 4), which sets it apart from all previously reported *p*-type redox-active organic materials [36]. In general, taking into account the low- and high-potential redox reactions, the *peri*-substituted disulfides can be categorized as bipolar compounds exhibiting simultaneously *n*- and *p*-type interactions at around 2.1 and 4.3 V vs. Li/Li^+^.

To rationalize the different kinetics of low- and high-redox reactions, the CV curves are analyzed based on the dependence of the current (i) on the scan rate (v). By increasing the scan rate from 1 mV/s to 100 mV/s, the current of both low- and high-voltage bands increases obeying a *v*^1/2^-dependence. This evidence the occurrence of diffusion-controlled reactions in low- and high-voltage regions. From the slope of the observed dependence (di/d*v*^1/2^), the diffusion coefficient, D, can be estimated using the Randles–Ševčík equation [38]; at 25 °C, the *D* can be expressed by D = [(di/d*v*^1/2^)/(2.69 × 10^5^ n^3/2^ A C)]^2^, where *n* is the number of electrons, *C* reflects the charge concentration, and *A* is the electrode surface area. In order to avoid the uncertainties in defining the real values for n, C, and A parameters, we used the relative diffusion coefficient with an aim to compare more accurately the effect of the halogen substituents and the alkyl chain length on the redox reactions of *peri*-derived compounds: *D*/*D*_SCl4_ = (di/d*v*^1/2^)^2^/(di/d*v*^1/2^)^2^_SCL4_, where *D*_SCl4_ is used as a reference value. The calculated values are given in Figure 4. Two features can be highlighted. First, the diffusion coefficient for high-voltage redox reaction exceeds more than two times that of the low-voltage reaction. This means that the high-voltage redox reaction (classified as *p*-type) proceeds faster than the low-voltage redox reaction classified as *n*-type. The established different kinetics for high- and low-voltage redox reactions are in good agreement with previous findings on *p*- and *n*-type organic electrode materials [36,39]. On the other hand, this supports once again the assignment of high-voltage redox reactions to oxidation of *peri*-substituted compounds followed by bonding with TFSI^−^ electrolyte anions, while the low-voltage redox reaction comes from the reduction of the sulfur moiety, including reversible cleavage and formation of the S–S bond by Li^+^ cations.

**Figure 5:**
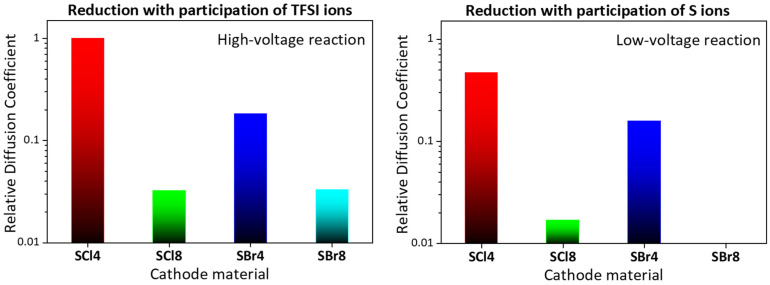
Relative diffusion coefficient for high- and low-voltage redox reactions ((**left**) and (**right**), respectively) determined from CV curves.

Furthermore, the comparison of data discloses that the highest diffusion coefficient is reached for the chloro-substituted compound with a short alkyl chain (Figure 5). In the high-voltage region, the diffusion coefficient decreases more than one order by extending the alkyl chain from 4 to 8. This trend is obeyed for the low-voltage redox reaction too (Figure 5). Both high- and low-voltage reactions exhibit slower kinetics when Cl atoms are replaced with Br atoms, this dependence is better expressed for the compounds with shorter alkyl chain. These interesting findings need further theoretical investigations.

**Figure 6:**
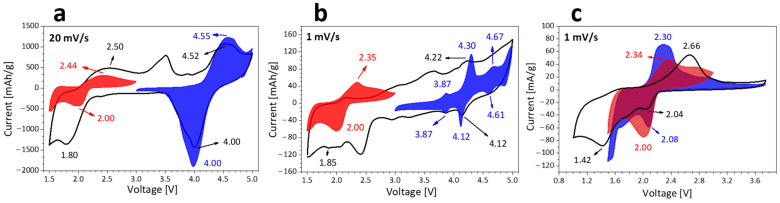
CV curves of **SCl4** recorded in low-voltage window between 1.5–3.0 V and high-voltage window between 3.0–5.0 V at a scan rate of 20 mV/s (**a**) and 1 mV/s (**b**). For the sake of comparison, the CV curve in an extended voltage window between 1.5 and5.0 V is also given. CV curves recorded in a potential range of 1.5–3.0 V (red), 1.5–3.75 V (blue), and 1.0–3.75 V (black lines) (**c**).

The next issue related to the redox properties of *peri*-substituted compounds is whether the CV curve profile depends on the potential limits where the redox reactions take place. Figure 6 compares the CV curve profiles of the **SCl4** recorded in two narrower potential regions: between 1.5 and 3.0 V and between 3.0 and 5.0 V. For the sake of better comparison, the CV curve profiles recorded in an extended potential range between 1.5 and 5.0 V are also given. As one can see, the high-voltage band (due to the *p*-type redox reaction) retains its shape and position in narrow and broad potential regions. It is worth mentioning that the high-voltage band becomes better resolved when the slower scan rate and narrower potential limits are used. In this case, the broad band is split into several peaks at 4.67/4.61 V, 4.30/4.12 V, and 3.87/3.87 V, thus suggesting a proceeding of multi-centre redox reaction. Contrary to the high-voltage redox reaction, the low-voltage reaction (due to the *n*-type interaction) is strongly dependent on the potential limits: in a narrow potential range between 1.5 and 3.0 V, one broad wave is more clearly resolved than the wave appearing after the recording a curve in an extended range of 1.5–5.0 V. This indicates that the *n*-type reaction undergoes some change (in terms of the activation of –C=O in addition to –S−S− redox interaction with Li^+^) if the *p*-type redox reaction occurs. This change also reflects the reaction kinetics: the relative diffusion coefficient decreases by more than one order when the reaction proceeds in narrow potential range. However, the E_1/2_ remains nearly the same: E_1/2_ = 2.14 V, 2.17 V, 2.19 V, and 2.04 V for the potential limits of 1.5–5.0 V, 1.5–3.0 V, 1.5–3.75 V, and 1.0–3.75 V (Figure 6c).

## 4. Conclusions

This study proposes a new concept for designing organosulfur compounds with regulated redox properties. As a proof-of-concept, we designed the *peri*-disulfo-substituted 1,8-naphthalimide derivatives with a fixed disulfide bridge, an added imide acceptor fragment, and an alkyl chain with a different length, as well as the presence of Cl or Br atoms at positions 3 and 6. The compounds are synthesized via a nucleophilic substitution of the halogen atoms at both *peri*-positions with elemental sulfur in a very-high yield and purity.

The redox properties of the new compounds were studied in model Li-ion cells with liquid ionic electrolyte. All compounds simultaneously display *n*- and *p*-type redox reactions between 1.5 and 5.0 V: at about 2.0 V vs. Li/Li^+^, there is a reversible reduction of the sulfur bridge with Li^+^; while above 4.0 V vs. Li/Li^+^, the oxidation of the organic compound followed by a binding with TFSI^−^ anions occurs. The high-voltage reaction is faster than the low-voltage one. The redox potentials of the *p*- and *n*-type redox reactions are mainly sensitive to whether Cl or Br substituents are available in the molecule architecture, while the alkyl chain length determines the kinetics of the redox reactions. As a result, the chloro-substituted compound with the shorter alkyl chain displays the best kinetics for both low- and high-voltage redox reactions.

In general, this study is the first report of peri-dithiolo-1,8-naphthalimides as bipolar organic redox compounds. Among them, the compound with the shorter alkyl chain and Cl-substitution (i.e., **SCl4**) appears to be the most appropriate candidate for electrochemical applications. Because of the big difference in the potentials of low- and high-voltage redox reactions, the reported organosulfur compounds present an interesting option for application as electrodes in symmetrical ion cells, which presents a new perspective in the future development of full organic ion batteries. In addition, the established potential of about 4.3 V vs. Li/Li^+^ for SCl4 makes it an interesting option as an electrode in dual-ion batteries, which presents a new direction in the utilization of redox organic compounds.

## Data Availability

Data are contained within the article.

## Supplementary Materials

The following supporting information can be downloaded at https://www.mdpi.com/article/10.3390/ma16237471/s1: Figure S1: ^1^H NMR and ^13^C NMR spectra of **3**–**6** in CDCl_3_; Figure S2: ^1^H NMR and ^13^C NMR spectra of **SCl8** in CDCl_3_; Figure S3: ^1^H NMR and ^13^C NMR spectra of **SBr8** in CDCl_3_; Figure S4: ^1^H NMR and ^13^C NMR spectra of **SCl4** in C_2_D_2_Cl_4_; Figure S5: ^1^H NMR and ^13^C NMR spectra of **SBr4** in C_2_D_2_Cl_4_.
