# Supplementary material for: Naphthalene Monoimides with Peri-Annulated Disulfide Bridge—Synthesis and Electrochemical Redox Activity
Source: Materials (Basel). 2023 Dec 1;16(23):7471. doi: 10.3390/ma16237471 (PMC10707240; doi:10.3390/ma16237471)
Supplement: Supplementary file 1 [file materials-16-07471-s001.zip › materials-2733544-supplementary.pdf]

materials

MDPI

Supplementary materials

Naphthalene monoimides with *peri*-annulated disulfide bridge – synthesis and electrochemical redox activity

Monika Mutovska [1], Natali Simeonova [1], Stanimir Stoyanov [1], Yulian Zagranyarski [1,*] Silva Stanchovska [2], Delyana Marinova [2,*]

[1] Faculty of Chemistry and Pharmacy, Sofia University "St. Kliment Ohridski", 1164 Sofia, Bulgaria
[2] Institute of General and Inorganic Chemistry, Bulgarian Academy of Sciences, 1113 Sofia, Bulgaria
* Correspondence: manasieva@svr.igic.bas.bg (D.M); ohjz@chem.uni-sofia.bg (Y.Z.)

Table of figures:

Figure S1. ^{1}H NMR and ^{13}C NMR spectra of **3-6** in $CDCl_3$. 5
Figure S2. ^{1}H NMR and ^{13}C NMR spectra of **SCl8** in $CDCl_3$. 6
Figure S3. ^{1}H NMR and ^{13}C NMR spectra of **SBr8** in $CDCl_3$. 7
Figure S4. ^{1}H NMR and ^{13}C NMR spectra of **SCl4** in $C_2D_2Cl_4$. 8
Figure S5. ^{1}H NMR and ^{13}C NMR spectra of **SBr4** in $C_2D_2Cl_4$. 9

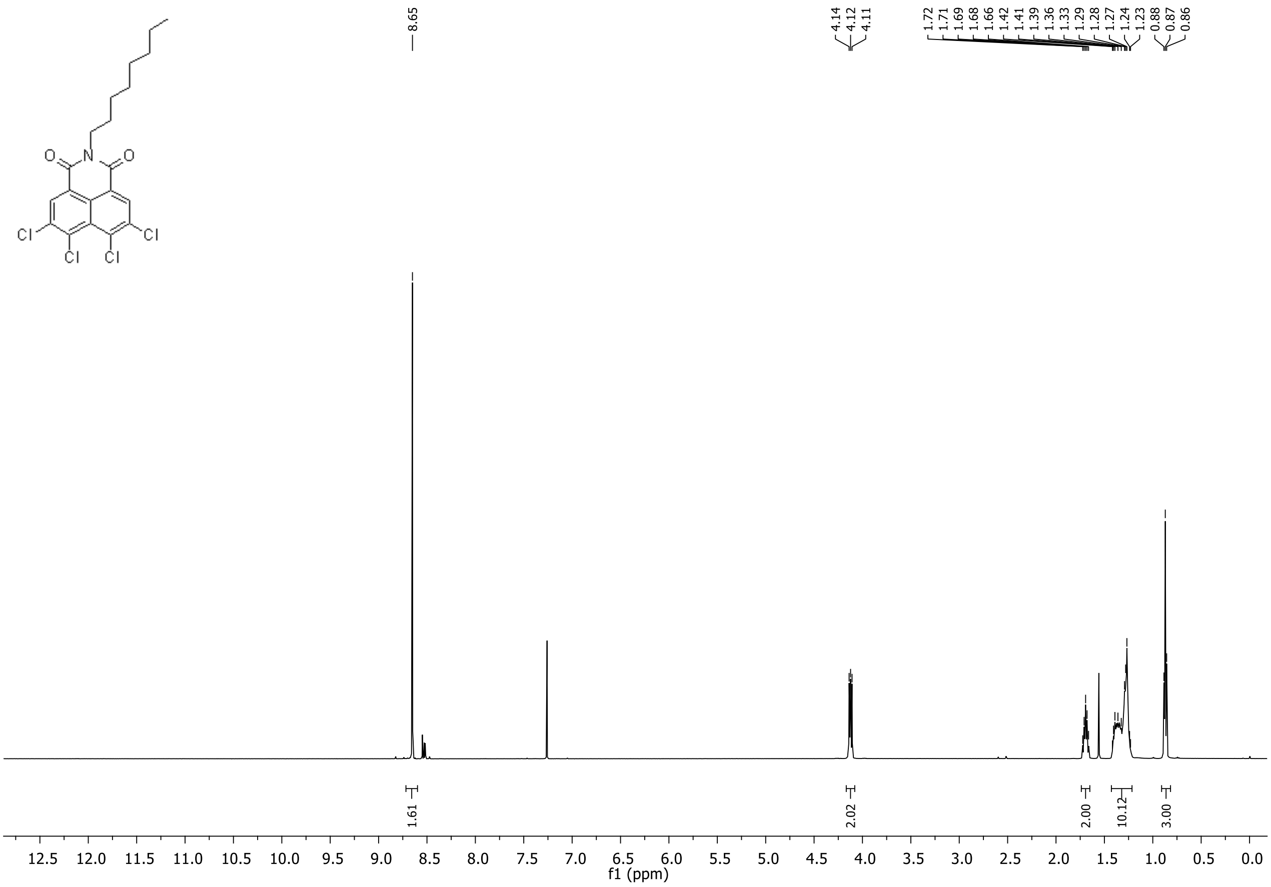

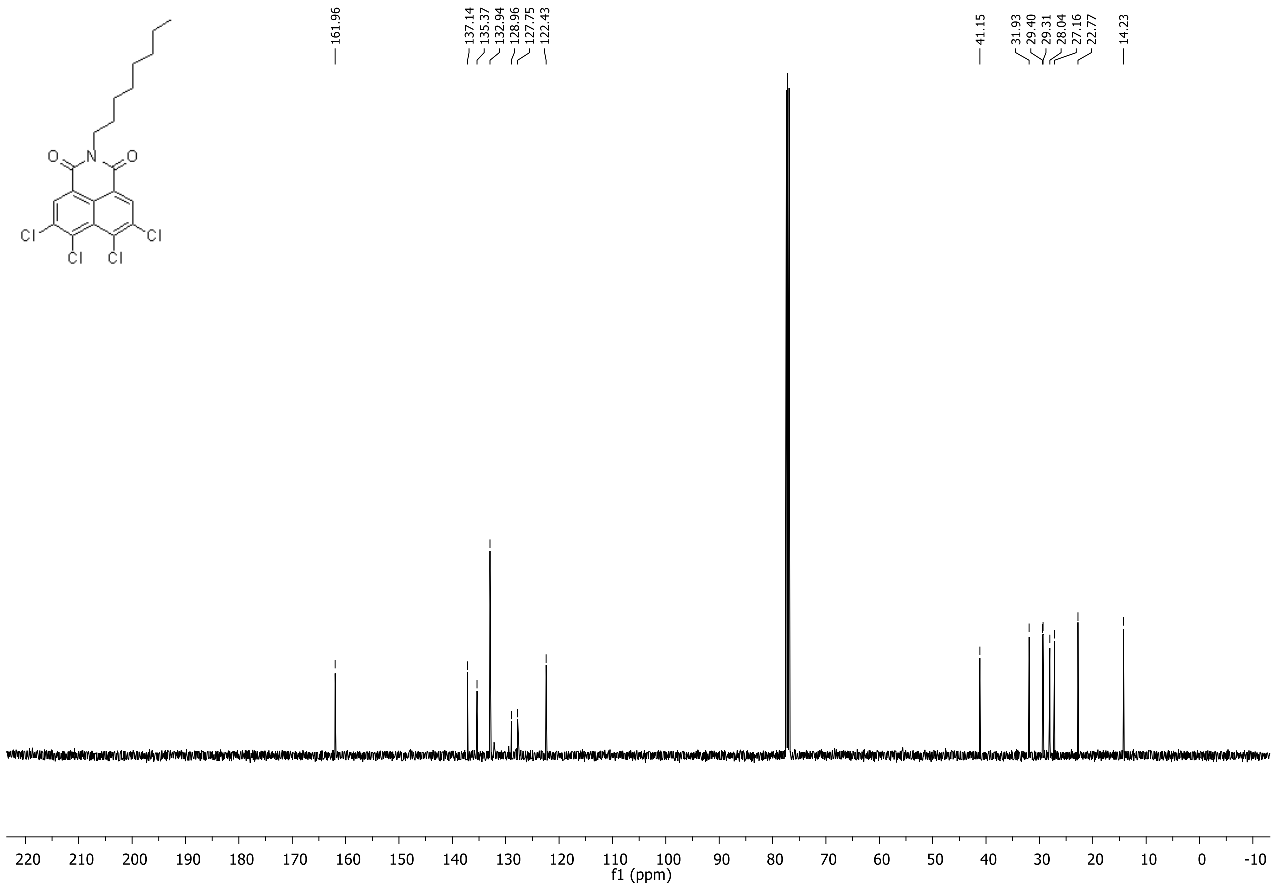
O
N
O
Cl
Cl
Cl
Cl
161.96
137.14
135.37
132.94
128.96
127.75
122.43
41.15
31.93
29.40
29.31
28.04
27.16
22.77
14.23
220 210 200 190 180 170 160 150 140 130 120 110 100 90 80 70 60 50 40 30 20 10 0 -10
f1 (ppm)

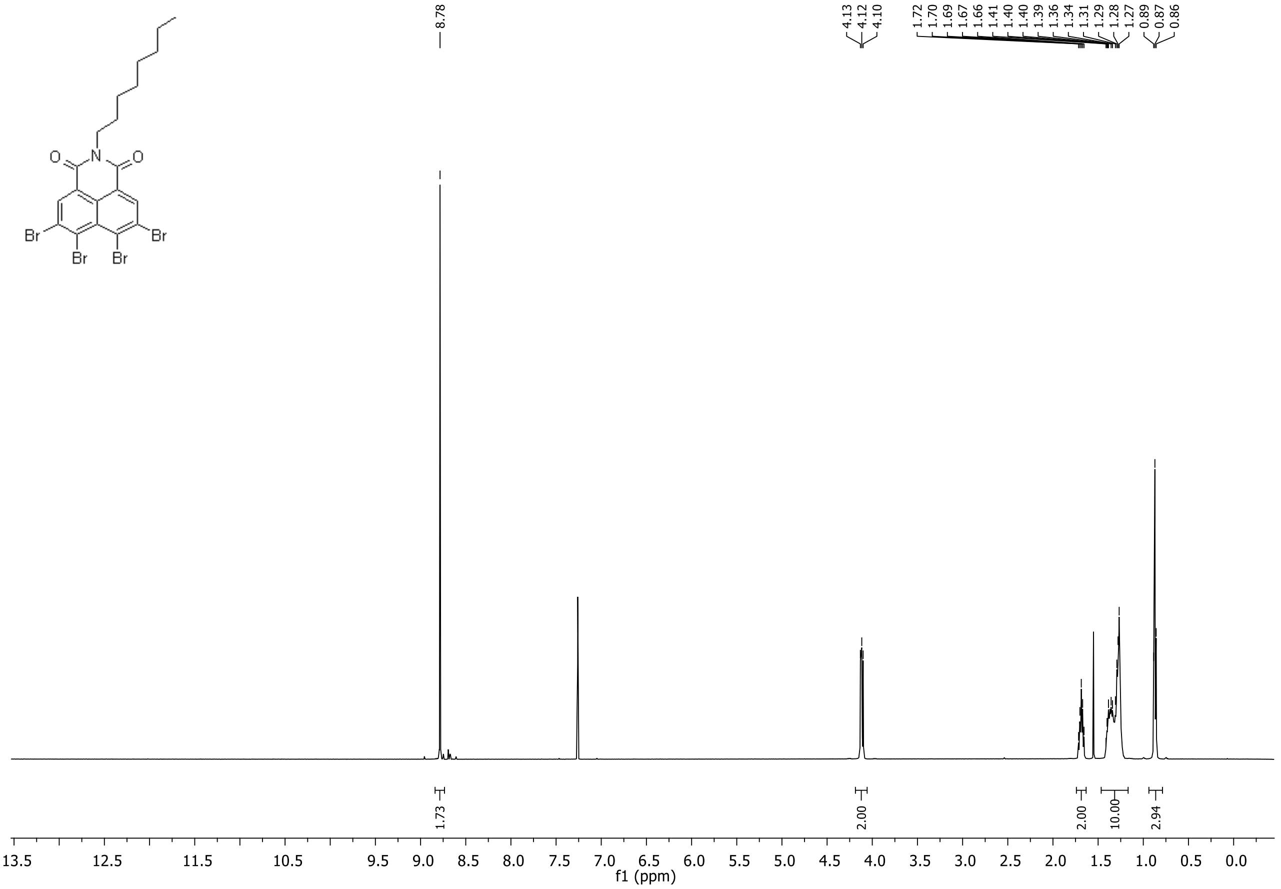
O
N
O
Br
Br
Br
Br
8.78
4.13
4.12
4.10
1.72
1.70
1.69
1.67
1.66
1.41
1.40
1.40
1.39
1.36
1.34
1.31
1.29
1.28
1.27
0.89
0.87
0.86
1.73
2.00
2.00
10.00
2.94
13.5 12.5 11.5 10.5 9.5 9.0 8.5 8.0 7.5 7.0 6.5 6.0 5.5 5.0 4.5 4.0 3.5 3.0 2.5 2.0 1.5 1.0 0.5 0.0
f1 (ppm)

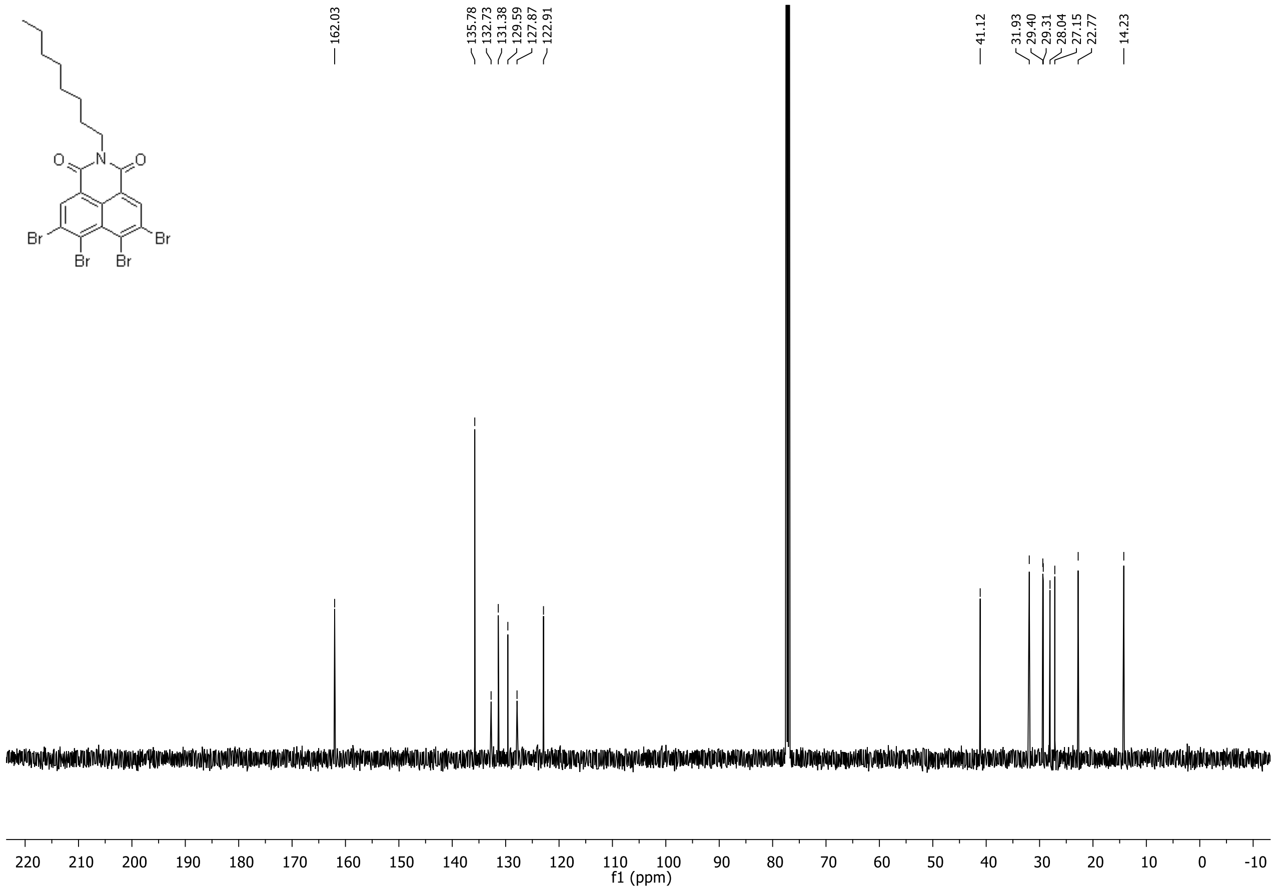
162.03
135.78
132.73
131.38
129.59
127.87
122.91
41.12
31.93
29.40
29.31
28.04
27.15
22.77
14.23
O
N
O
Br
Br
Br
Br
220 210 200 190 180 170 160 150 140 130 120 110 100 90 80 70 60 50 40 30 20 10 0 -10
f1 (ppm)

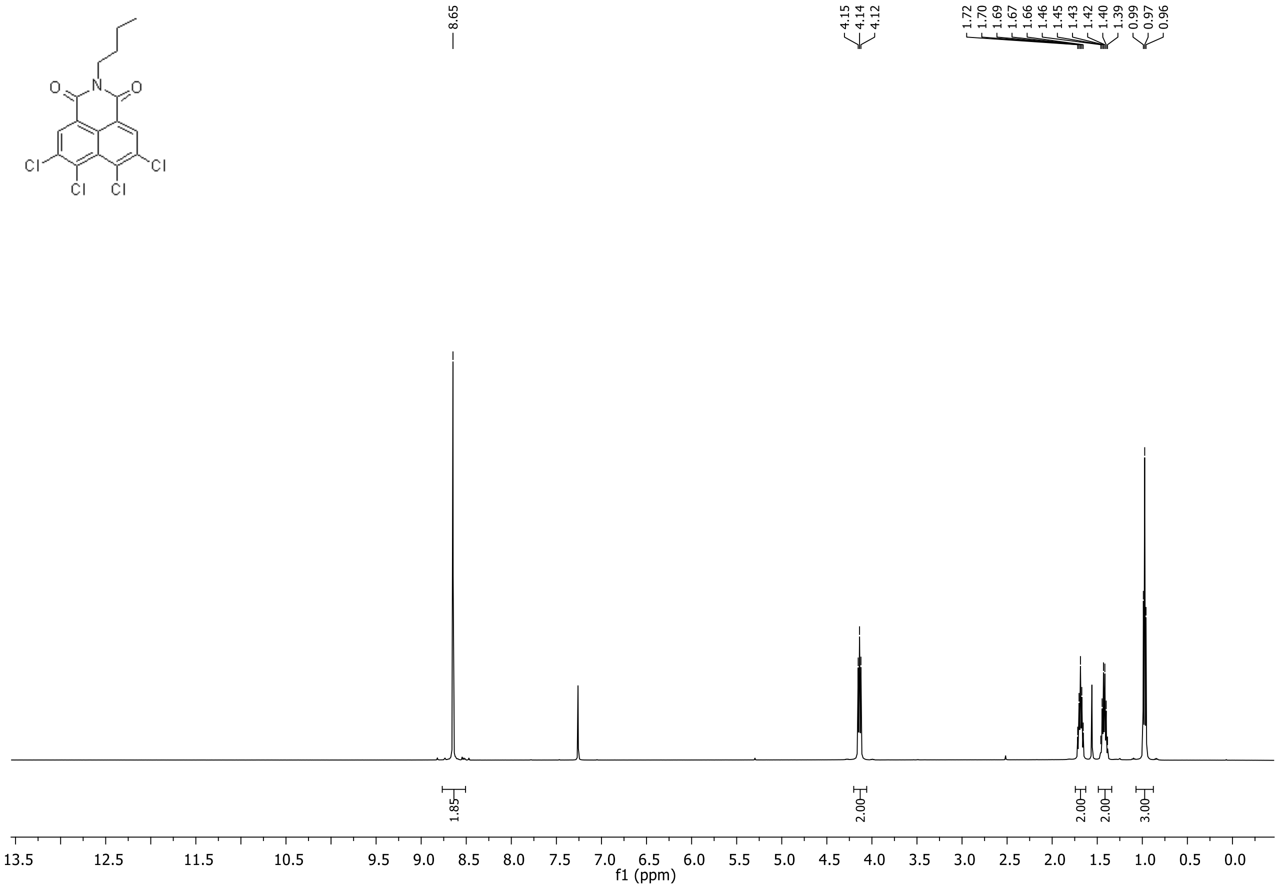
8.65
4.15
4.14
4.12
1.72
1.70
1.69
1.67
1.66
1.46
1.45
1.43
1.42
1.40
1.39
0.99
0.97
0.96
O
N
O
Cl
Cl
Cl
Cl
1.85
2.00
2.00
2.00
3.00
13.5 12.5 11.5 10.5 9.5 9.0 8.5 8.0 7.5 7.0 6.5 6.0 5.5 5.0 4.5 4.0 3.5 3.0 2.5 2.0 1.5 1.0 0.5 0.0
f1 (ppm)

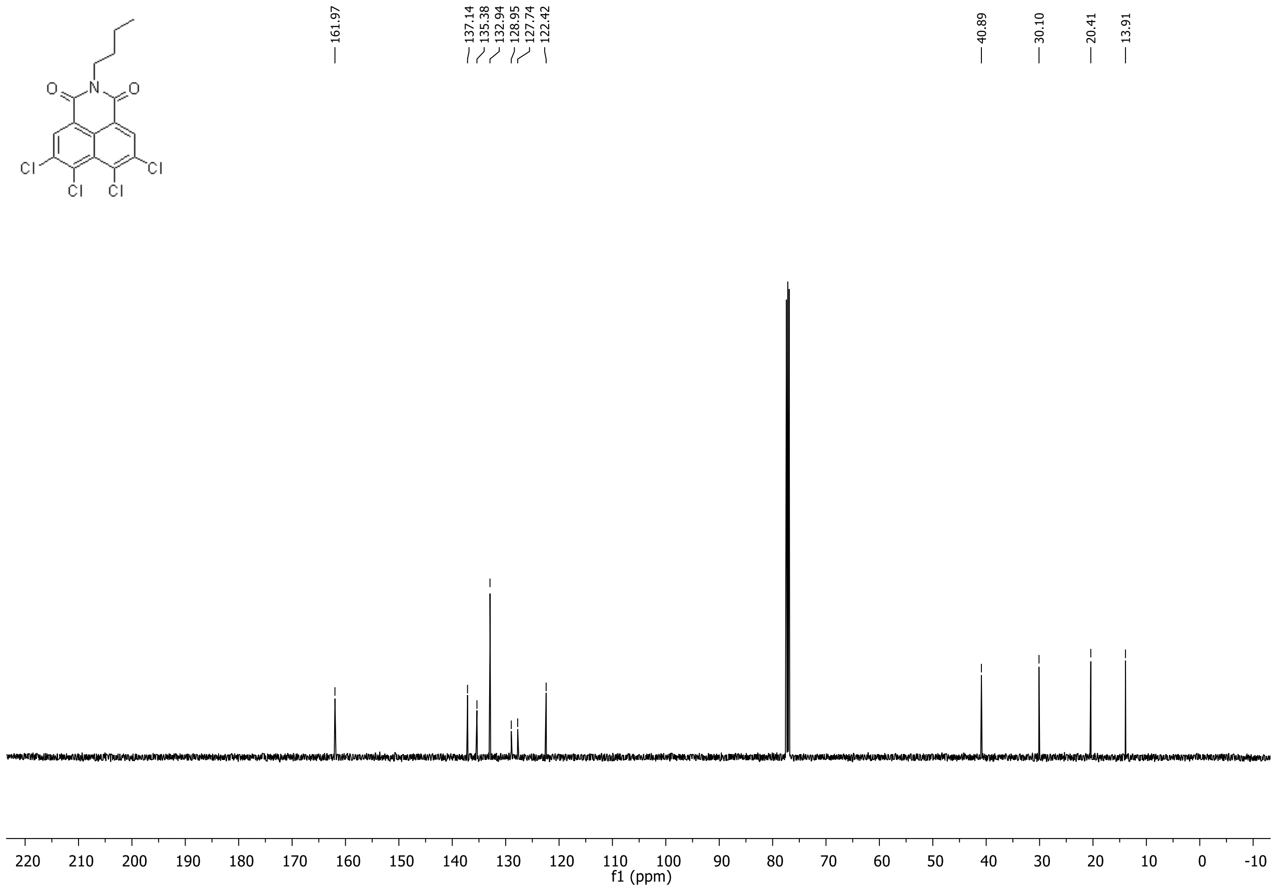

O
N
O
Cl
Cl
Cl
Cl
161.97
137.14
135.38
132.94
128.95
127.74
122.42
40.89
30.10
20.41
13.91
220 210 200 190 180 170 160 150 140 130 120 110 100 90 80 70 60 50 40 30 20 10 0 -10
f1 (ppm)

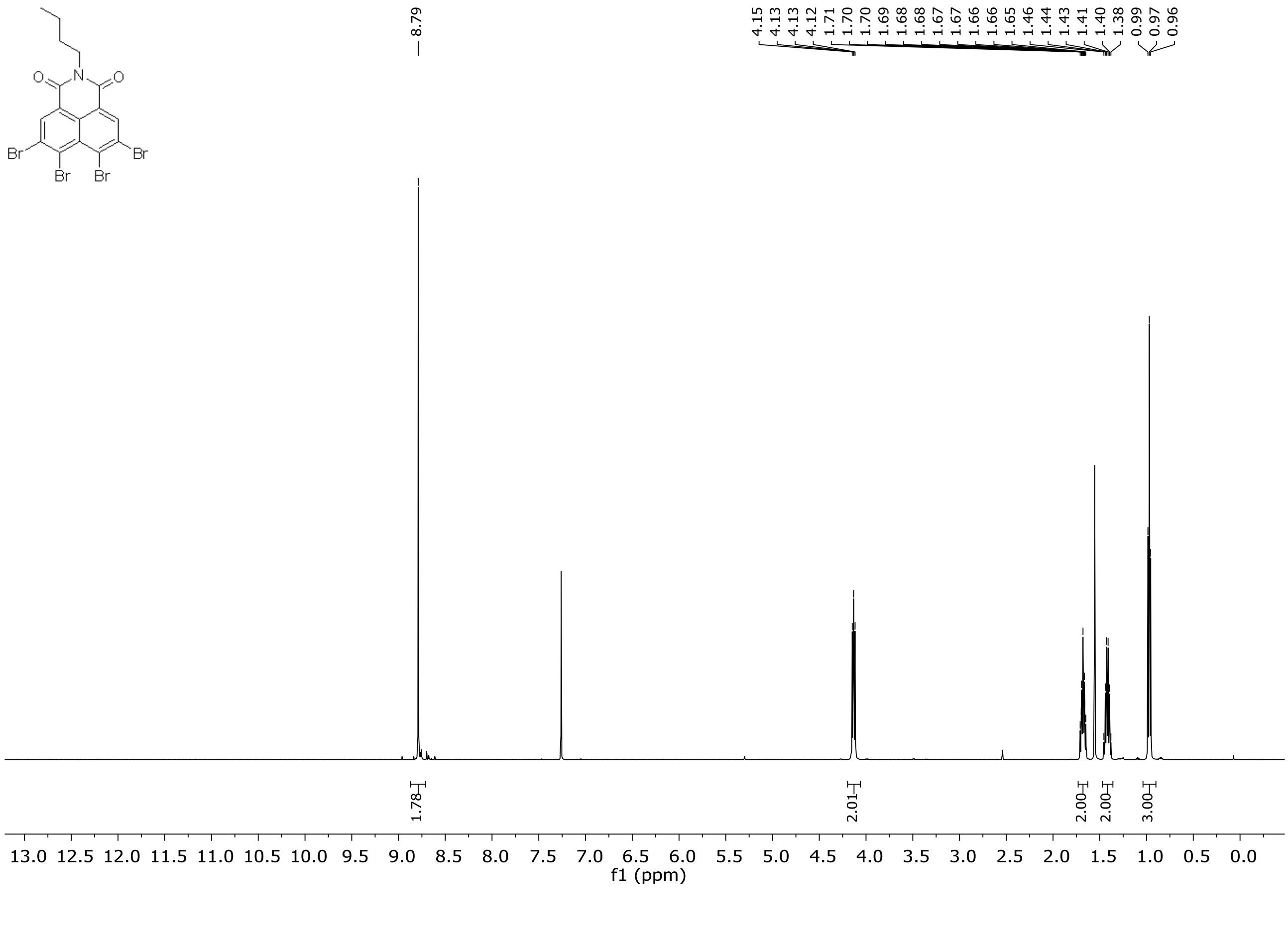

O
N
O
Br
Br
Br
Br
8.79
4.15
4.13
4.13
4.12
1.71
1.70
1.70
1.69
1.68
1.68
1.67
1.66
1.66
1.65
1.46
1.44
1.43
1.41
1.40
1.38
0.99
0.97
0.96
1.78
2.01
2.00
2.00
3.00
13.0 12.5 12.0 11.5 11.0 10.5 10.0 9.5 9.0 8.5 8.0 7.5 7.0 6.5 6.0 5.5 5.0 4.5 4.0 3.5 3.0 2.5 2.0 1.5 1.0 0.5 0.0
f1 (ppm)

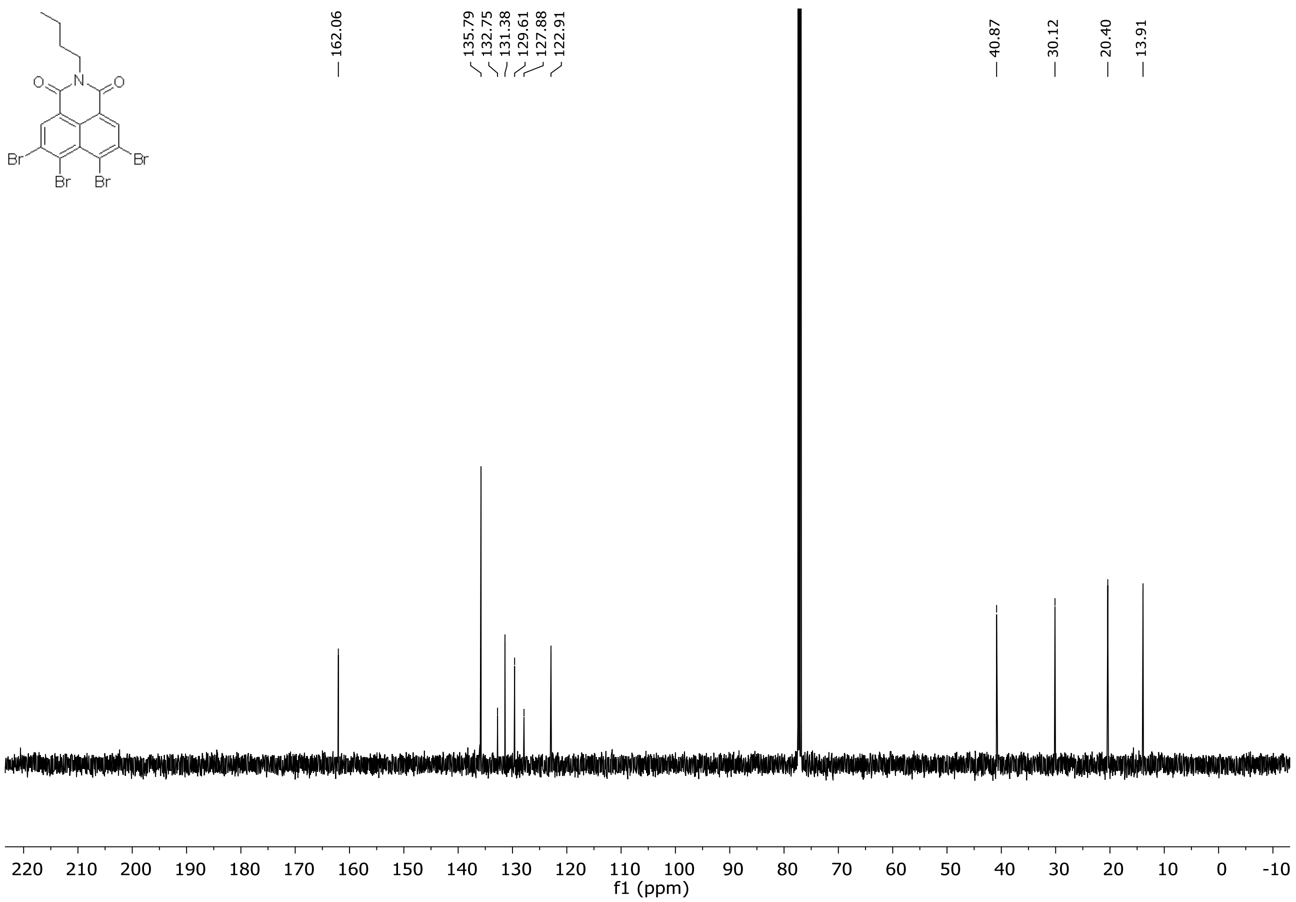

Figure S1. ^{1}H *NMR and* ^{13}C *NMR spectra of* ***3-6*** *in* $CDCl_3$*.*

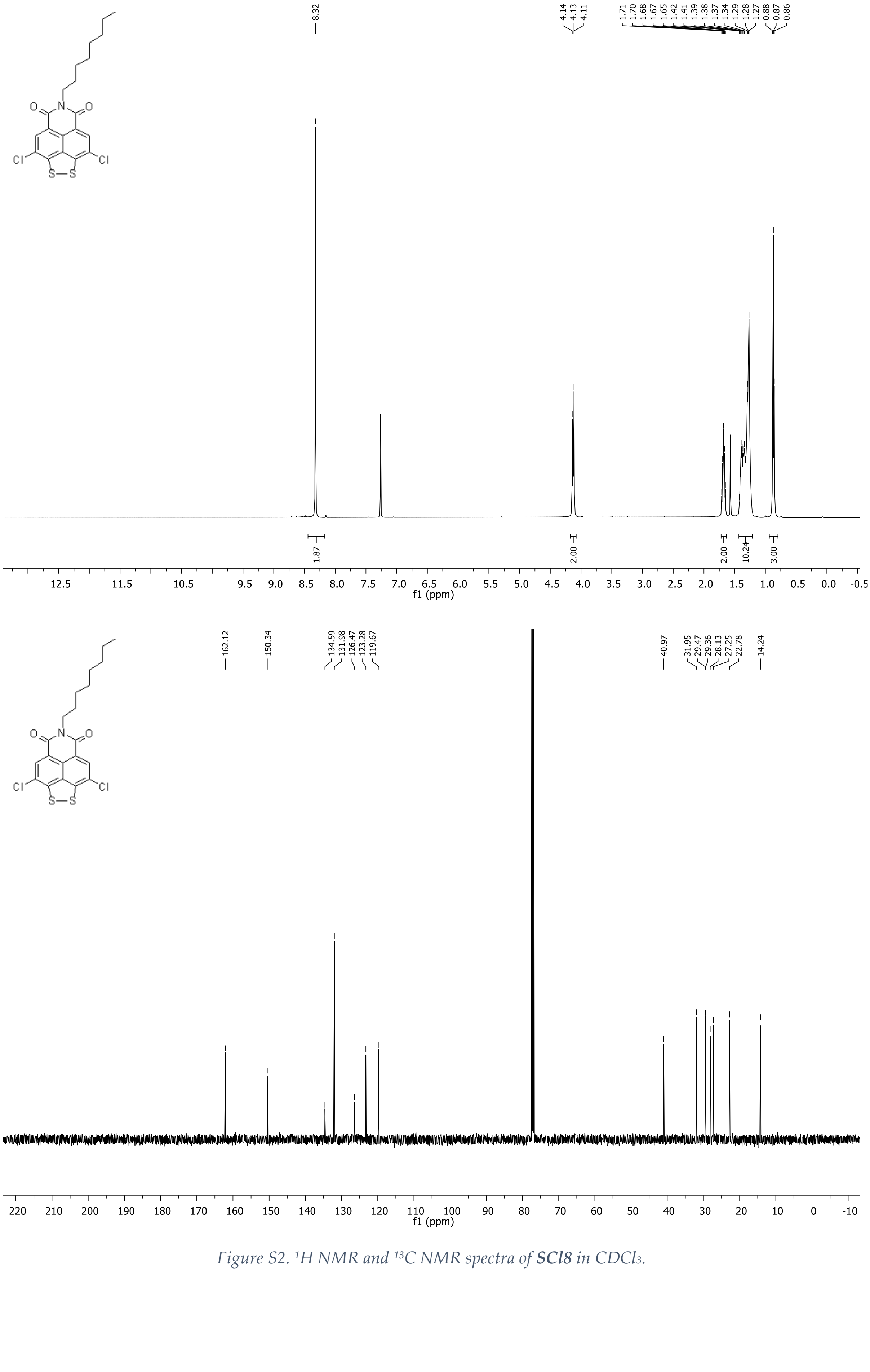

Figure S2. ^{1}H NMR and ^{13}C NMR spectra of ***SCl8*** *in* $CDCl_3$*.*

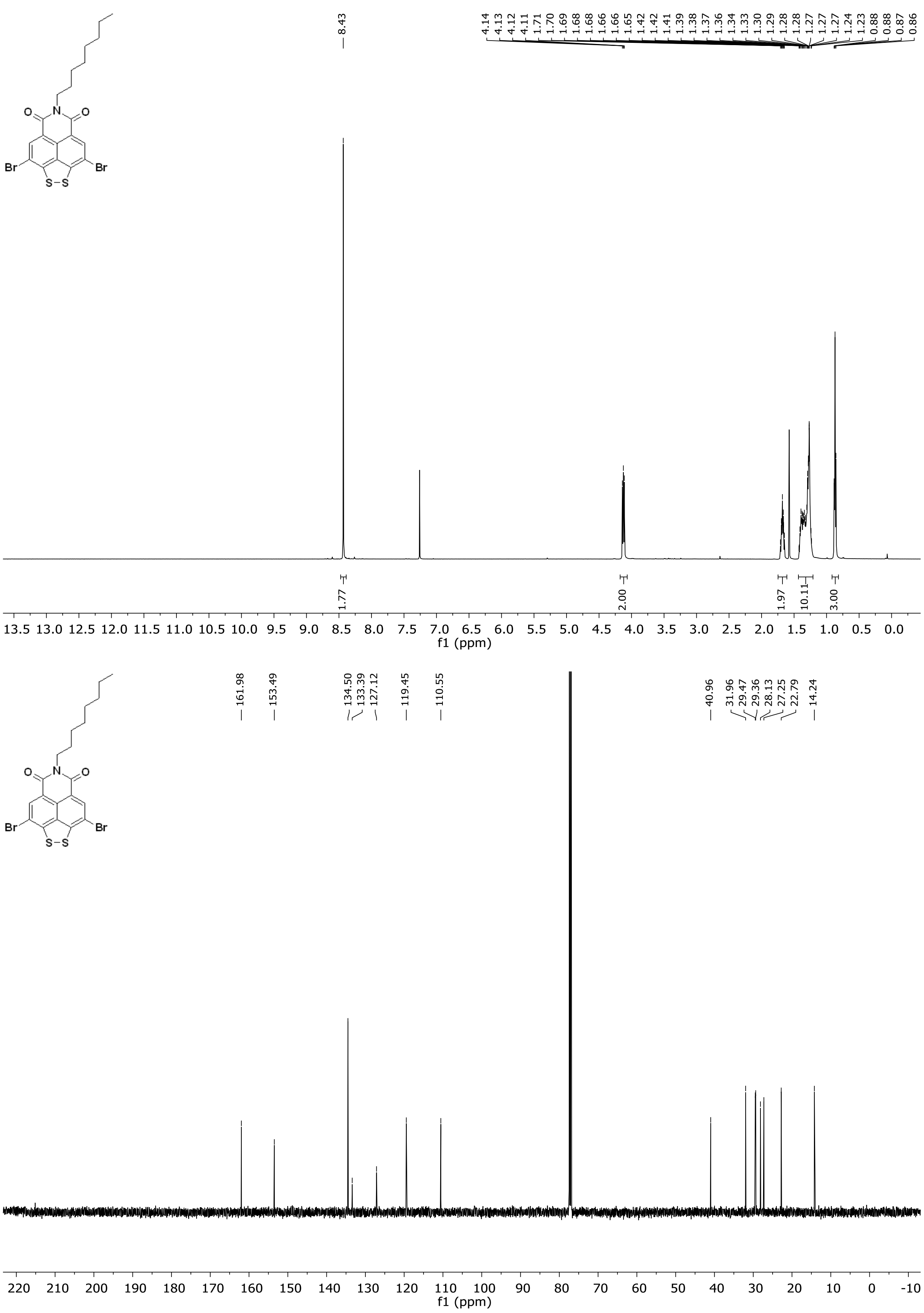

Figure S3. ^{1}H NMR and ^{13}C NMR spectra of ***SBr8*** *in $CDCl_3$.*

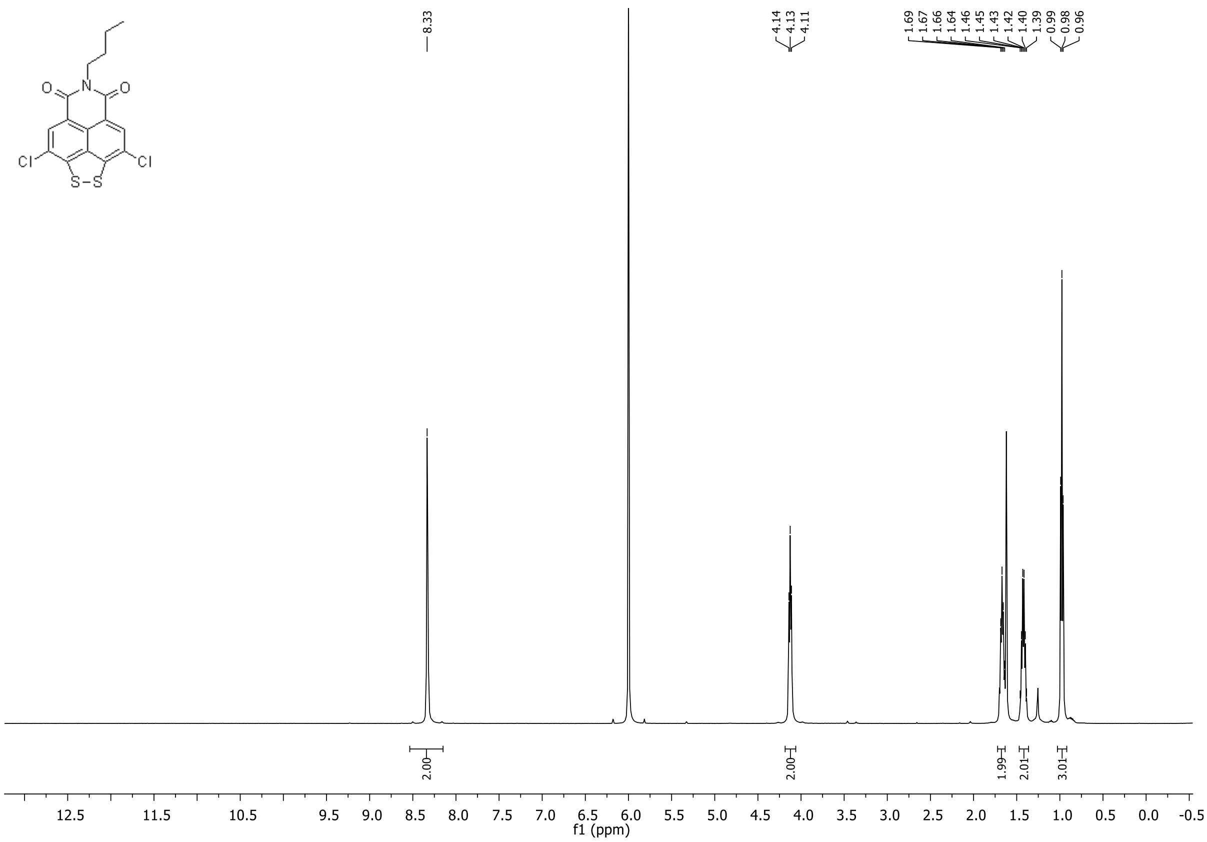

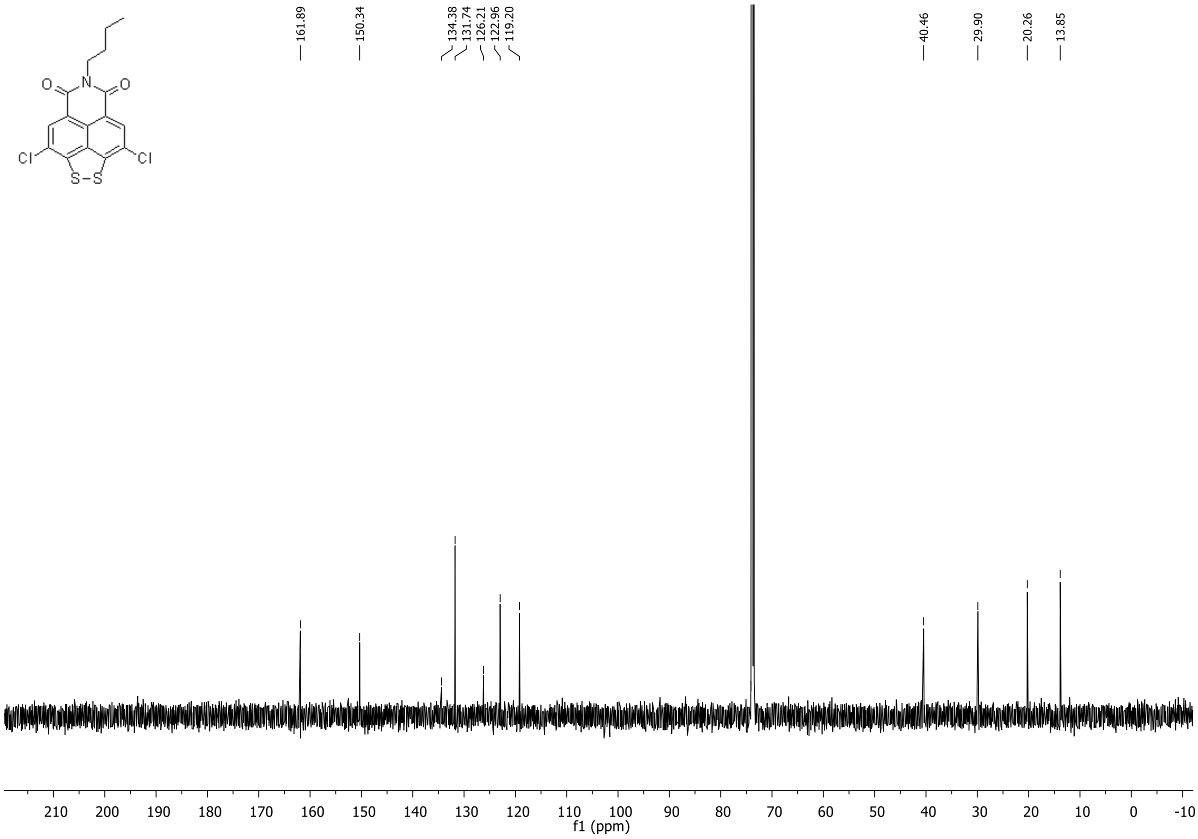

Figure S4. ^{1}H *NMR and* ^{13}C *NMR spectra of* ***SCl4*** *in* $C_2D_2Cl_4$.

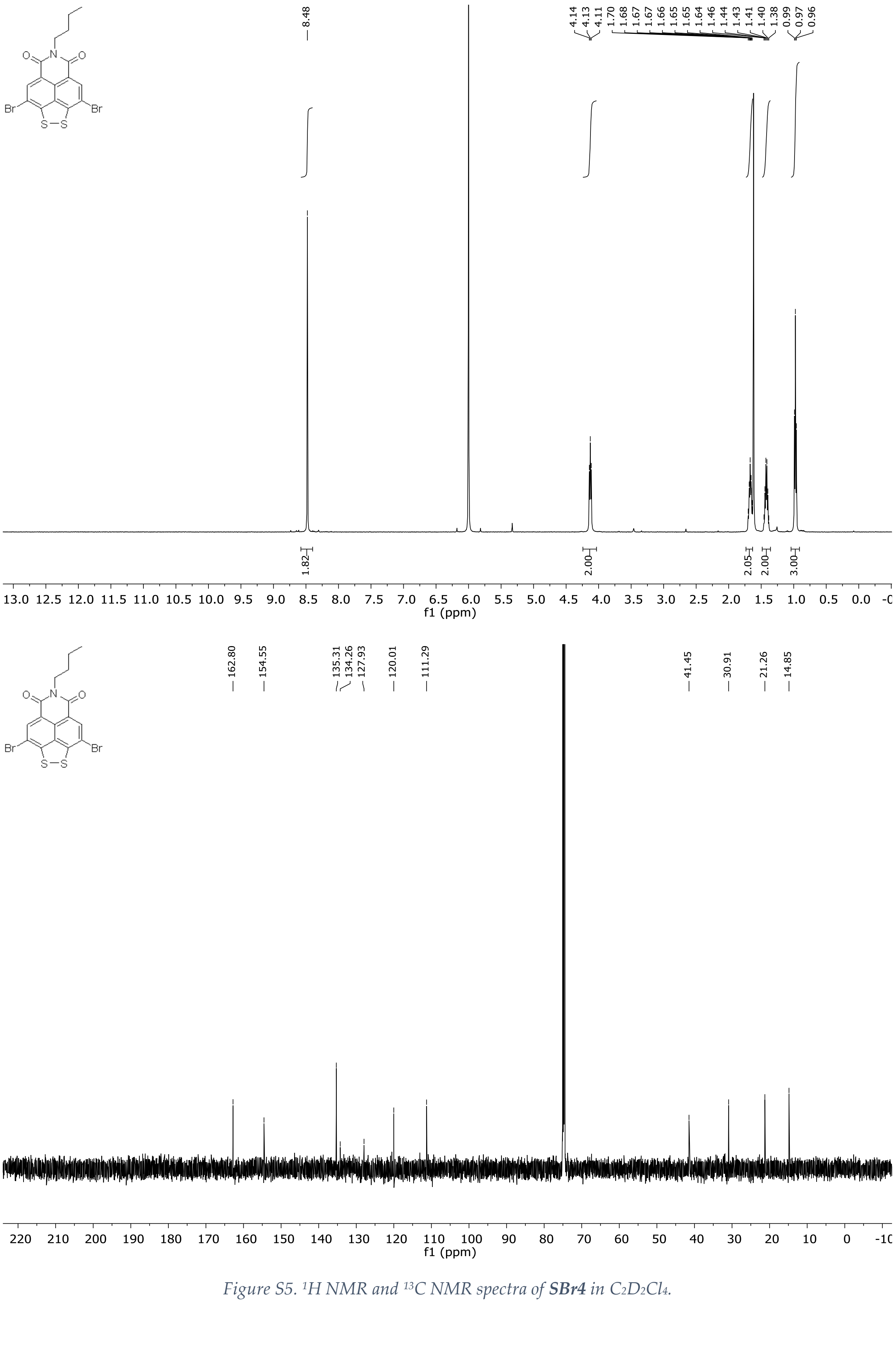

*Figure S5. ^{1}H NMR and ^{13}C NMR spectra of **SBr4** in $C_2D_2Cl_4$.*
